# *Fusarium* spp. and *Aspergillus flavus* infection induces pathogen-specific and pathogen-independent host immune response in patients with fungal keratitis

**DOI:** 10.3389/fcimb.2025.1560628

**Published:** 2025-05-30

**Authors:** Shreya Dinesh, Lalitha Prajna, Prajna Namperumalsamy Venkatesh, Kuppamuthu Dharmalingam, Bharanidharan Devarajan

**Affiliations:** ^1^ Department of Microbiology and Bioinformatics, Aravind Medical Research Foundation, Madurai, India; ^2^ Department of Biomedical Sciences, Madurai Kamaraj University, Madurai, India; ^3^ Department of Ocular Microbiology, Aravind Eye Hospital, Madurai, India; ^4^ Cornea and Refractive Surgery Services, Aravind Eye Hospital, Madurai, India; ^5^ Department of Proteomics, Aravind Medical Research Foundation, Madurai, India

**Keywords:** fungal keratitis (FK), differential gene expression, pathogen specific host immune response, IL-17 signalling pathway, RT-qPCR analysis

## Abstract

**Introduction:**

Fungal keratitis, caused primarily by *Fusarium* spp. and *Aspergillus flavus*, is a significant cause of corneal blindness, particularly in tropical regions. Current antifungal agents like natamycin and voriconazole have limited efficacy, underscoring the need for a deeper understanding of host immune responses.

**Methods:**

This study employed high-throughput RNA sequencing to investigate differential gene expression in human corneal tissues from patients with *Fusarium* spp. and *A. flavus* keratitis and compared them to control cadaver corneal samples. RNA was extracted from infected and control samples, followed by sequencing and differential expression analysis. Further confirmation of differential expression of selected genes were carried out by Real-Time quantitative PCR (RT-qPCR).

**Results:**

Data analysis identified common and *Fusarium* spp. and *A. flavus*-specific differentially expressed genes (DEGs). Pathway enrichment analysis using common genes identified pathways enriched in both infections, such as interleukin 17 (IL-17), tumor necrosis factor (TNF), and chemokine signalling. Expression of hub genes, including S100 calcium binding protein A7 (S100A7), S100 calcium binding protein A8 (S100A8), S100 calcium binding protein A9 (S100A9) and C-X-C motif chemokine ligand 8 (CXCL8), identified in interleukin 17 (IL-17) signalling, was confirmed by RT-qPCR analysis. *Fusarium* spp.-specific DEGs, including complement C3 (C3), interleukin 6 (IL-6), interleukin 19 (IL-19) and leucine rich alpha-2-glycoprotein 1 (LRG1), are enriched in pathways such as positive regulation of immune responses, acute inflammatory responses, leukocyte cell-cell adhesion, and the regulation of cell-cell adhesion. *A. flavus*-specific DEGs, such as triggering receptor expressed on myeloid cells 2 (TREM2) and apolipoprotein E (APOE), are predominantly enriched in adaptive immune response, negative regulation of immune system process, negative regulation of immune response, cell migration and motility pathways.

**Discussion:**

RT-qPCR confirmed the key pathogen-specific DEGs, highlighting their potential as biomarkers for pathogen-specific immune responses. These findings provide insights into the distinct immune pathways triggered by *Fusarium* spp. and *A. flavus*, offering new therapeutic targets for improving fungal keratitis treatment.

## 1 Introduction

Fungal keratitis (FK) is a significant cause of ocular morbidity in tropical parts of the world (Atta et al., 2022). *Fusarium* spp. and *Aspergillus flavus* are responsible for about 95% of fungal corneal infections (Brown et al., 2021). Clinical presentation typically includes symptoms such as intense pain, blurred vision, redness, excessive tearing, and photophobia. If untreated, FK can progress to corneal ulceration, opacification, and, in severe cases, endophthalmitis, resulting in permanent vision loss (Brown et al., 2021). The treatment of FK mainly relies on antifungal drugs natamycin and voriconazole; however, the efficacy of these treatments is often suboptimal, particularly for infections caused by *A. flavus* and *Fusarium* spp. (Venkatesh Prajna et al., 2013). The Mycotic Ulcer Treatment Trial (MUTT) highlighted the importance of identifying the specific fungus responsible for the infection in order to guide appropriate treatment, and this study also showed that natamycin is more effective for the treatment of *Fusarium* spp. keratitis, while voriconazole is more effective for treating *Aspergillus* spp. infections (Venkatesh Prajna et al., 2013). Despite these treatments, prognosis remains poor, prompting recent interest in immune system-targeting therapies, which have shown potential in improving patient outcomes (Abbondante et al., 2023; Suman et al., 2024).

A deeper understanding of the immune response mechanisms during fungal infection, particularly regarding the host’s defense against *A. flavus* and *Fusarium* spp., is essential to improve the management of fungal keratitis. Several studies have highlighted pathogen-specific differences in host immune responses to these pathogens—for example, Mallela et al. (2021) reported a reduced expression of methyl-CpG binding domain protein 3 (mBD3) during *A. flavus* infection. In contrast, an increase in mBD3 expression was observed in *Fusarium solani* infection (Kolar et al., 2013). Additionally, Kolar et al. (2013) found that the absence of mBD3 in mice exacerbated symptoms during *Fusarium solani* infection, suggesting its role in immune defense. Shait Mohammed et al. (2020) reported the absence of Factor H-like (FHL) protein in the tears of patients with *Fusarium* spp. keratitis, while it was present in *A. flavus* keratitis patients’ tear. Similarly, the expression of zinc alpha-2 glycoprotein (ZAG) level was progressively decreased in *A. flavus* patients’ tears, unlike in *Fusarium* spp. infection (Ananthi et al., 2013; Parthiban et al., 2019).

These findings highlight the species-specific immune responses that influence the course of the disease. Building on these observations, we hypothesized that the pathophysiology of *Fusarium* spp. and *A. flavus* keratitis differs due to distinct immune activation pathways tailored to each pathogen’s unique characteristics. To explore this, we employed an unbiased transcriptional profiling approach to investigate the host immune response in human corneal tissue from *A. flavus* and *Fusarium* spp. keratitis patients. Our study aimed to identify differentially expressed genes involved in pathogen-specific and pathogen-independent host immune responses, which could provide a better understanding of the host response in fungal keratitis.

## 2 Materials and methods

### 2.1 Samples

The study included fungal keratitis samples collected from patients undergoing therapeutic penetrating keratoplasty (TPK) at Aravind Eye Hospital, Madurai. Corneal tissues were collected post-surgery and stored at -80°C. Fungal identification was carried out at two key stages. First, at the patient’s initial presentation, scraping of corneal ulcer was performed under aseptic precautions, and two smears were prepared on glass slides for 10% potassium hydroxide (KOH) wet mount and Gram staining. A microscopic examination of these smears confirmed the presence of fungal filaments. In addition, material from the scraping was directly inoculated onto potato dextrose agar and incubated at 25°C for 7–14 days. Based on colony morphology and microscopic examination, the fungus was identified as either *A. flavus* or *Fusarium* spp (Gunasekaran et al., 2021). In *A. flavus*-infected cases, fungal identification confirmed the presence of *A. flavus* in all patients. However, for *Fusarium* spp., species-level identification was not performed, as it is not a possible routine diagnostic procedure. The culture was confirmed as a true causative agent only if the organism was grown at the area of streaking, and thus contamination and co-culture were ruled out. Second, following TPK, a small portion of corneal tissue was used to reconfirm the presence of the fungal organism using the same protocol as mentioned above. Only patients for whom both the initial and post-TPK identifications confirmed *A. flavus* or *Fusarium* spp. were included in the study. Samples that tested positive for bacterial contamination or mixed infection were excluded from the study. For mRNA sequencing, five samples were collected from each infection group (*A. flavus* and *Fusarium* spp.), with additional five samples per group for real-time quantitative PCR (RT-qPCR) validation. A total of 20 control cadaver corneas were obtained from Rotary Aravind International Eye Bank, trephined immediately, snap-frozen in liquid nitrogen, and stored at -80°C. The mean age of the patients was 53.5 years (± 12.7), while the control group’s mean age was 65.8 years (± 11.4) (**Supplementary Table S1**). The exclusion criteria included acute or chronic systemic illness, topical steroid therapy, or any form of immunosuppression. All participants presented unilateral corneal infections and provided written informed consent prior to sample collection. The samples were collected from patients during the period 2022–2023, and the diagnosis rates for *Fusarium* spp. and *A. flavus* were 43.05% and 15.5%, respectively, for this period. This study adhered to the Declaration of Helsinki guidelines and was approved by the Institutional Review Board, Aravind Medical Research Foundation, Madurai, India (IR82020008BAS).

### 2.2 Extraction of RNA

Normal donor corneas (*n* = 10) and post-TPK corneas culture-positive for *Fusarium* spp. (*n* = 5) and *A. flavus* (*n* = 5) were used for mRNA sequencing. Briefly, corneal tissues stored in Trizol were thawed and transferred to a 2-mL microcentrifuge tube for homogenization. Chloroform was added to homogenized samples, and the aqueous phase was separated by centrifugation at 12,000 RPM for 15 min. This step was repeated for a better quality of RNA. Then, RNA was precipitated in isopropanol. Pellets were washed in 75% ethanol, air-dried, and eluted in nuclease-free water. Initially, RNA quantity and quality were assessed by the absorbance ratio at 260/280 nm using a NanoDrop 2000 spectrophotometer (Thermofisher Scientific, DE, UK). Samples with an RNA integrity number greater than 7.0, as evaluated using an RNA nano-chip in a bioanalyzer (RNA ScreenTape System (catalog: 5,067–5,576) in a 4150 TapeStation System (catalog: G2992AA; Agilent Technologies, Waldbronn, Germany) were used for library preparation.

### 2.3 Library construction of cDNA and mRNA sequencing

QIAseq Stranded RNA Library Kit (Qiagen, cat. no. 180753) was used to create mRNA libraries according to the manufacturer’s instructions. An improved reverse transcription enzyme and a buffer system were used for first-strand cDNA synthesis following RNA fragmentation. A specific enzyme mixture and a buffer formulation were used to synthesize the second cDNA strand with A-base overhangs, facilitating the effective ligation of Illumina-compatible adapters. The Clean Start PCR mix amplified the RNA-seq libraries, ensuring strong amplification across regions with high GC or AT content. Sequencing was performed at Biokart Private Limited, Bangalore, India (BIOPROJECT PRJNA1171184).

### 2.4 mRNA sequencing data analysis

**Figure 1:**
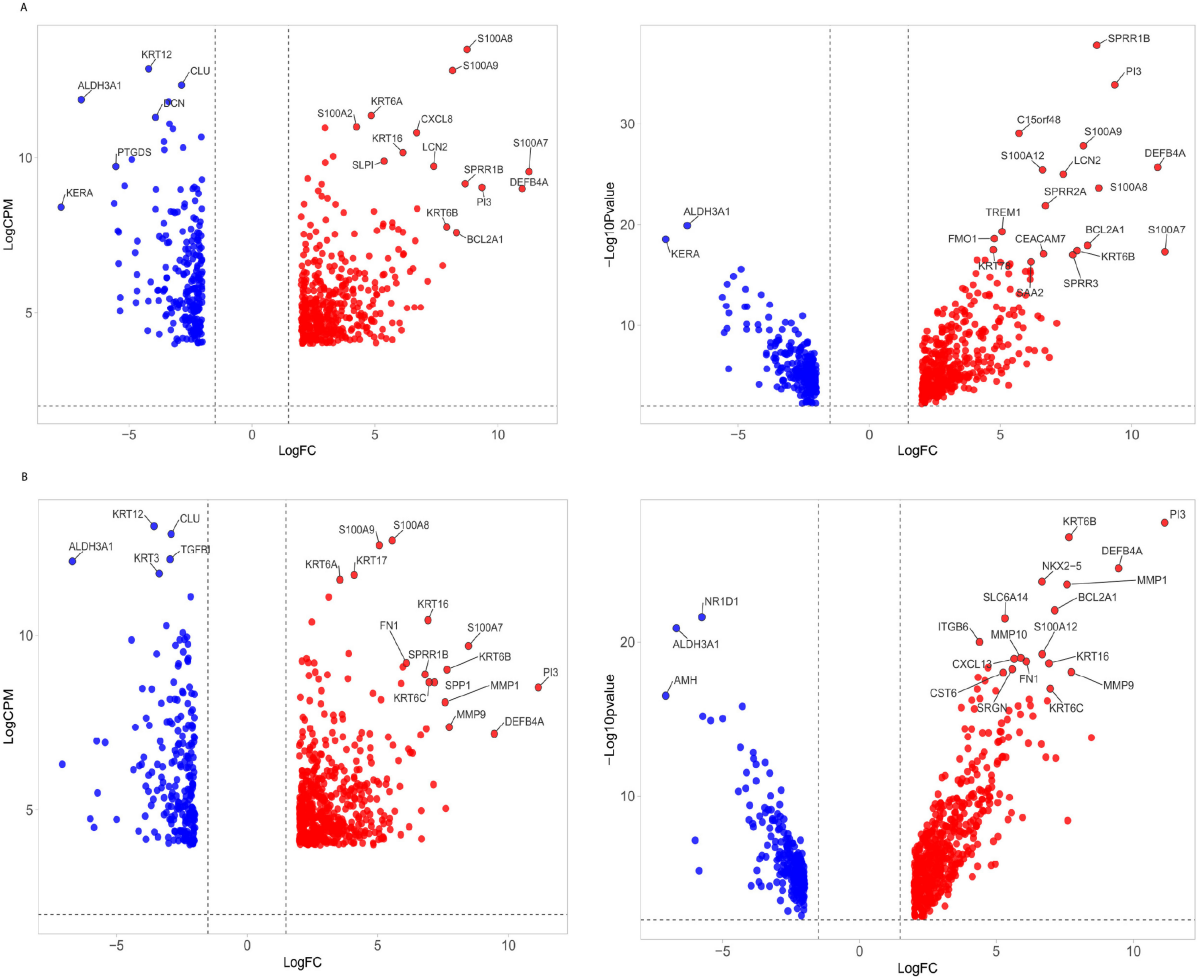
Volcano plot of differentially expressed genes (DEGs) in fungal keratitis. **(A)** The volcano plot illustrates the distribution of DEGs in corneal tissues infected with *Fusarium* spp. and **(B)** *A. flavus* compared to control samples. Left panel: The x-axis represents the log2 fold change (log2FC), while the y-axis shows the logCPM. Right panel: Log10 adjusted *p*-value. Genes with significant upregulation (log2FC ≥ 2, FDR < 0.05) are marked in red, while significantly downregulated genes (log2FC ≤ -2, FDR < 0.05) are marked in blue. The top list of DEGs is labeled.

**Figure 2:**
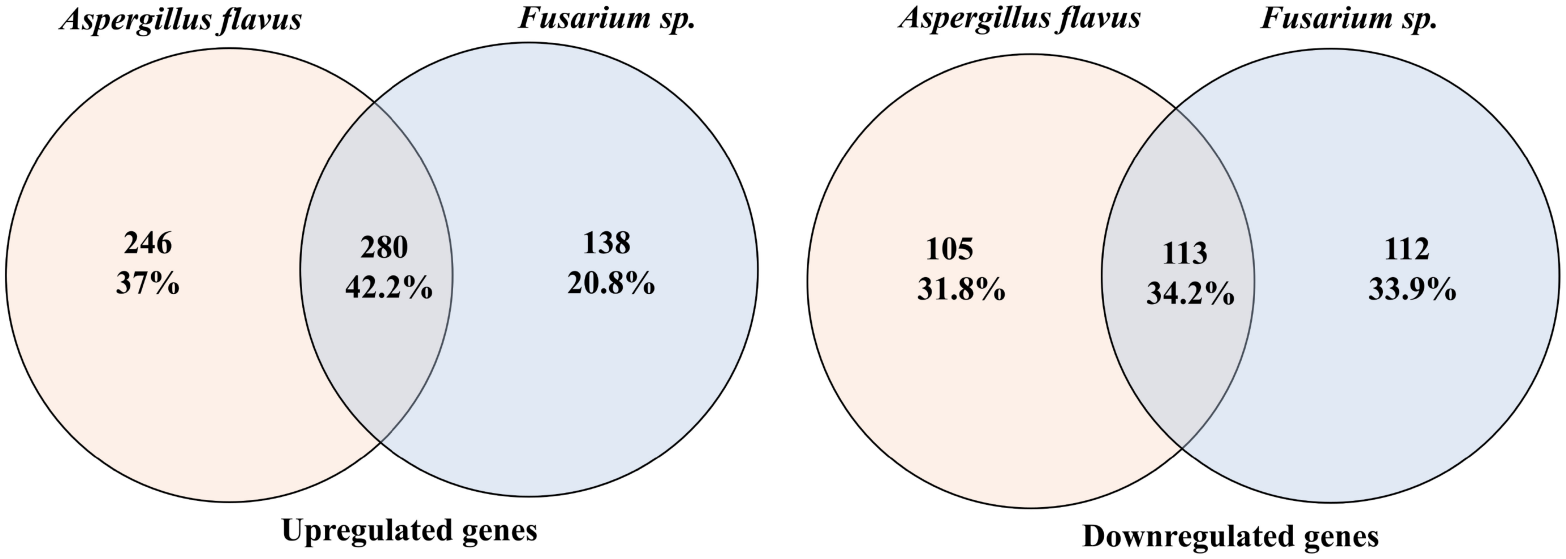
Venn diagrams comparing differentially expressed genes in *Fusarium* spp. and *A. flavus* infection. Left: Venn diagram for upregulated genes. Right: Venn diagram for downregulated genes.

Raw deep sequencing data were obtained in the FASTQ format and assessed for quality using FastQC version 0.11.8 (Smith and de Sena Brandine, 2021). Adapter sequences and low-quality reads shorter than 50 nucleotides were filtered out with Fastp (Chen et al., 2018). The reads were aligned to the human reference genome GRCh38 using STAR version 2.4.0.1 (Dobin et al., 2013), and quantification was carried out with Feature Counts (Liao et al., 2014) using Ensembl release 104 (Yates et al., 2020). Differential expression (DE) analysis was conducted with DEApp (https://yanli.shinyapps.io/DEApp/) (Li and Andrade, 2017) to compare control samples with those infected by *A. flavus* and *Fusarium* spp. The input data included a raw count data file with summarized counts for all samples and a meta-data Table with experimental design details for each sample. Features with low expression (CPM ≤ 1 in fewer than two samples) were removed post-alignment. The data were then normalized, and DE analysis was performed using edgeR with thresholds set at log fold change (log FC) > 1.5 and a false discovery rate (FDR) adjusted *P*-value < 0.05. VolcaNoseR (Goedhart and Luijsterburg, 2020) was used to create volcano plots, displaying log2 fold change versus log2 CPM (**Figure 1**) and log2 fold change versus -log10*P*-value (**Figure 1**). Differentially expressed genes (DEGs) were filtered with the criteria of log2 fold change (FC) <−2 or >2 and logCPM ≥4. We identified common and pathogen-specific DEGs using Venn diagram tool Venny (https://bioinfogp.cnb.csic.es/tools/venny/) (**Figure 2**).

### 2.5 Pathway enrichment analysis

Pathway enrichment analyses were performed on common and pathogen-specific DEGs using Bioconductor R-package clusterProfiler version 3.18.1 (Yu et al., 2012). Enrichment analyses for Gene Ontology (GO) functions and Kyoto Encyclopedia of Genes and Genomes (KEGG) pathways were carried out using the gseGO and gseKEGG functions from the clusterProfiler package. They were selected as significant if the threshold was less than 0.05.

### 2.6 Pathway–gene network analysis for identification of hub genes

The functional network was plotted using Cytoscape version 3.9.1 (Shannon et al., 2003) for GO terms and KEGG pathways enriched using common and pathogen-specific DEGs. The GO terms analysis, which identified more than 100 genes, was then prioritized based on *p*-value with a cutoff of 0.01. The list of GO terms was then compared between common and *A. flavus* and *Fusarium* spp. groups to identify pathogen-specific GO terms. These selected pathways were then merged based on the similarity in gene sets using the R tool GOsemsim (Yu et al., 2010). Hub genes were identified using Cytoscape’s CytoHubba plugin, employing the betweenness centrality ranking method. The top 25 genes were selected as the cutoff.

### 2.7 Weighted gene co-expression network analysis

Weighted gene co-expression network analysis (WGCNA) was conducted separately on RNA-seq data from five *A. flavus*-infected samples, five *Fusarium* spp.-infected samples, and five cadaver cornea control samples for each infection group. Firstly, the hierarchical clustering utilized the Hclust function native to R to detect and exclude potential outliers. The pickSoftThreshold function was employed to determine an optimal soft-thresholding power, ensuring that the network adhered to a scale-free topology using the mean connectivity and *R* ^2^ correlation coefficient. Using the chosen soft-thresholding power, an adjacency matrix is established using the “adjacency” function. The adjacency matrix is then transformed into a topological overlapping matrix (TOM), considering the number of neighbors that the genes share. The corresponding dissimilarity matrix is computed from the TOM for subsequent network analysis. Subsequently, the Pearson correlation matrix was computed, and genes were grouped into modules using hierarchical clustering alongside the dynamic tree-cut algorithm with a module eigengene dissimilarity threshold of 0.25. The traits matrix was created using the expressions of genes in infected samples and control samples. Gene significance (GS) and module membership (MM) were computed to correlate modules with traits. The genes that belong to significant modules were selected separately from *Fusarium* spp.-infected and *A. flavus*-infected samples for further downstream analysis. The selected genes from these modules of *Fusarium* spp. and *A. flavus* infection were compared using the online Venn analysis tool Venny to identify common and pathogen-specific genes. Furthermore, protein–protein interactions (PPIs) were obtained using STRING version 12.0 (https://string-db.org). The PPI network was constructed using Cytoscape, and hub genes were identified using cytohubba (**Supplementary Figure S1**).

### 2.8 Confirmation of selected DEGs using real time-quantitative PCR

Selected mRNAs were validated in 10 infected (five *A. flavus* and five *Fusarium* spp.) samples (post-TPK Tissue) and 10 control samples (cadaver cornea). RNA was isolated from all of the tissues using the conventional Trizol–chloroform RNA extraction method. Isolated RNA was quantified using the nanodrop quantification method and was reverse-transcribed into cDNA by using miscript RT kit. miscript SYBR Green RT-qPCR kit was used to quantify mRNAs using custom-synthesized forward and reverse primers. The primer sequences for all of the mRNAs are provided in **Supplementary Table S2**. The reaction conditions included an initiation step at 95°C for 10 min, followed by 40 cycles of 95°C for 10 s, 60°C for 1 min, and 72°C for 1 min. Each reaction was made in triplicates. The expression level of mRNAs was normalized with a reference control beta-ACTIN (*ACTB*). LogFC was calculated using the 2^-ddCT^ method.

## 3 Results

### 3.1 mRNA sequencing and differentially expressed genes

mRNA deep sequencing and principal component analysis (PCA) plot showed a clear segregation of infected samples from the control groups (**Supplementary Figure S2**). Further separation was observed between *A. flavus*- and *Fusarium* spp.-infected corneas, suggesting that there might be differential transcriptomic profiles between these two fungus-infected corneas (**Supplementary Figure S2C**). Differential expression analysis identified 2,544 and 2,651 differentially expressed genes (DEGs) in *A. flavus* and *Fusarium* spp. keratitis compared to cadaver control, respectively. After filtering based on log2 FC ≥ ± 2 and logCPM ≥ 4, we identified 744 DEGs in *A. flavus* samples and 643 DEGs in *Fusarium* spp. samples. Among the upregulated genes, 280 genes were shared, while 246 genes and 138 genes were uniquely upregulated in *A. flavus* and *Fusarium* spp., respectively. Similarly, 113 genes of the downregulated genes were common, with 105 genes being specific to *A. flavus* and 112 genes being specific to *Fusarium* spp., respectively (**Figure 2**).

### 3.2 Enriched functional pathways using common DEGs

**Figure 3:**
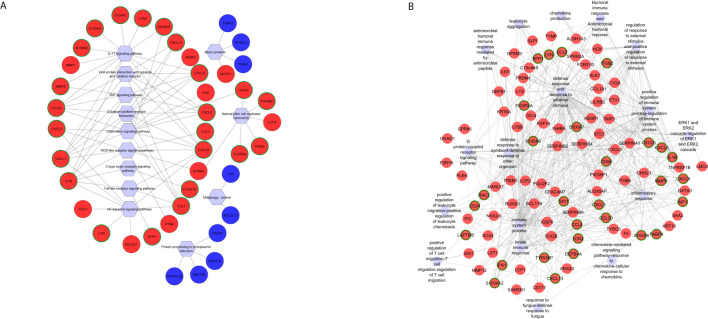
Functional networks of selected **(A)** KEGG pathways and **(B)** GO biological processes created using differentially expressed genes commonly identified in *Fusarium* spp. and *A. flavus* keratitis. Downregulated and upregulated genes are marked in blue and red, respectively. The hub genes are marked in green in outline. Hexagonal shapes represent the pathways or GO.

Functional enrichment analysis using common DEGs identified 28 KEGG pathways (**Supplementary Table S3**), including activated pathways like interleukin 17 (IL-17) signaling, chemokine signaling, cytokine–cytokine receptor interaction, and tumor necrosis factor (TNF) signaling and suppressed pathways like mitophagy—animal, protein processing in endoplasmic reticulum, and metabolic pathways (**Supplementary Table S3**). Hub gene analysis using CytoHubba highlighted six hub genes C-X-C motif chemokine ligand 8 (CXCL8), C-X-C motif chemokine ligand 5 (CXCL5), C-X-C motif chemokine ligand 6 (CXCL6), C-C motif chemokine ligand 4 (CCL4), C-X-C motif chemokine ligand 1 (CXCL1), and C-C motif chemokine ligand 20 (CCL20) shared between the cytokine–cytokine receptor and TNF signaling pathways (**Figure 3A**). Additionally, Rac family small GTPase 2 (RAC2) and C-X-C motif chemokine ligand 3 (CXCL3) were uniquely enriched in the chemokine signaling pathway, while CXCL8, interleukin 1 beta (IL-1B), and CCL4 were also involved in the toll-like receptor pathway (**Figure 3A**). Notably, eight genes were enriched in both the nuclear factor kappa-light-chain-enhancer of activated B cells (NF-kappa B) and nucleotide-binding oligomerization domain (NOD)-like receptor pathways, with CXCL8, IL-1B, CCL4, CXCL1, and CXCL3 as shared hub genes. Furthermore, six genes were enriched in the natural killer cell-mediated cytotoxicity pathway, with fc epsilon receptor Ig (FCER1G) identified as a hub gene and RAC2 specifically enriched in chemokine signaling and natural killer cell-mediated cytotoxicity (**Figure 3A**).

Gene Ontology (GO) enrichment analysis identified 232 biological processes (**Supplementary Table S4**). The top activated pathways were immune response, neutrophil chemotaxis, leukocyte chemotaxis, and top suppressed pathways including circadian regulation of gene expression, cellular response to starvation, and regulation of starvation (**Supplementary Table S4**). Immune response pathway enriched 89 genes, including 12 hub genes: C-X-C motif chemokine ligand 13 (CXCL13), allograft inflammatory factor 1 (AIF1), matrix metallopeptidase 12 (MMP12), IL-1B, S100 Calcium Binding Protein A7 (S100A7), CCL4, S100 Calcium Binding Protein A12 (S100A12), S100 Calcium Binding Protein A12 (S100A12), CCL20, S100 Calcium Binding Protein A8 (S100A8), CXCL8, and CXCL6 (**Figure 3B**). Among these, IL-1B was significantly enriched in 20 additional GO terms, such as inflammatory response, positive regulation of NF-kappa B transcription factor activity, and defense response (**Figure 3B**). Matrix metallopeptidase 9 (MMP9), another key hub gene, was enriched in inflammatory and defense response pathways, biological interaction between species, response to external stimuli, collagen catabolic processes, and cell migration (**Figure 3B**). Genes from the S100 family—S100A7, S100A12, S100A9, and S100A8—were enriched across pathways associated with neutrophil activation, neutrophil and granulocyte migration, leukocyte migration, cell migration, defense response to fungi, and responses to other organisms (**Figure 3B**). These genes were also identified as top hits in the volcano plot of differentially expressed genes (**Figure 1**), along with MMP9. The significance of hub genes, specifically IL-1B, MMP9, and S100 family members, in promoting inflammatory and immunological responses during fungal infections is highlighted by this investigation.

### 3.3 Pathogen-specific pathways in *Fusarium* spp. keratitis

**Figure 4:**
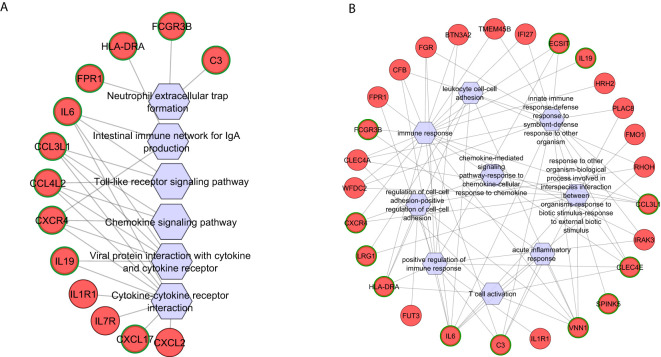
Functional networks of selected **(A)** KEGG pathways and **(B)** GO biological processes created using differentially expressed genes specific to *Fusarium* spp. keratitis. Upregulated genes are marked in red. The hub genes are marked in green in outline. Hexagonal shapes represent the pathways or GO.

KEGG pathway enrichment analysis of *Fusarium* spp.-specific DEGs identified 15 pathways, of which 13 were activated and two were suppressed (**Supplementary Table S3**), with functionally relevant ones selected for network analysis (**Figure 4A**). The top activated pathways included the viral protein interaction with cytokine and cytokine receptor pathway (five hub genes) and the chemokine signaling and toll-like receptor signaling pathways (three hub genes each). C-C motif chemokine ligand 3 like 1 (CCL3L1) and C-C motif chemokine ligand 4 like 2 (CCL4L2) were common to these three pathways, while C-X-C motif chemokine receptor 4 (CXCR4) overlapped between viral protein interaction and chemokine signaling pathways. C-X-C motif chemokine ligand 17 (CXCL17), enriched in the cytokine–cytokine receptor interaction pathway, highlighted its role in recruiting dendritic cells and macrophages for early host defense (Choreño-Parra et al., 2020). The neutrophil extracellular trap formation pathway was enriched with *Fusarium* spp.-specific DEGs complement C3 (C3), fc gamma receptor IIIb (FCGR3B), and formyl peptide receptor 1 (FPR1) (**Figure 4A**).

Gene Ontology (GO) enrichment analysis of *Fusarium* spp.*-*specific genes identified 87 biological processes (**Supplementary Table S4**), with the immune response pathway being significantly enriched, including 22 genes. Other significantly enriched, pathogen-specific pathways were the regulation of cell–cell adhesion, T-cell activation, and positive regulation of the immune response (**Figure 4B**). Seven hub genes vanin 1 (VNN1), interleukin 6 (IL-6), C-type lectin domain family 4 member E (CLEC4E), major histocompatibility complex, class II, DR alpha (HLA-DRA), C-C motif chemokine ligand 3 Like 1 (CCL3L1), C3, and CXCR4 were shared across multiple pathways, such as response to external biotic stimulus, regulation of intrinsic apoptotic signaling pathway, and interspecies interaction between organisms (**Figure 4B**). C3, a central regulator, was also enriched in innate immune response and immune response-regulating cell surface receptor signaling pathways. Pathways related to lymphocyte differentiation and activation enriched five and six genes, respectively, with CLEC4E, VNN1, IL-6, and HLA-DRA as shared hub genes (**Figure 4B**). The adaptive immune response pathway enriched five genes, with HLA-DRA identified as a key hub gene. Additionally, Leucine Rich Alpha-2-Glycoprotein 1 (LRG1), a regulator of an immune response, was enriched in pathways related to response to other organisms, interspecies interaction, regulation of leukocyte cell–cell adhesion, and immune cell adhesion mechanisms (**Figure 4B**).

### 3.4 Pathogen-specific pathways in *A. flavus* keratitis

**Figure 5:**
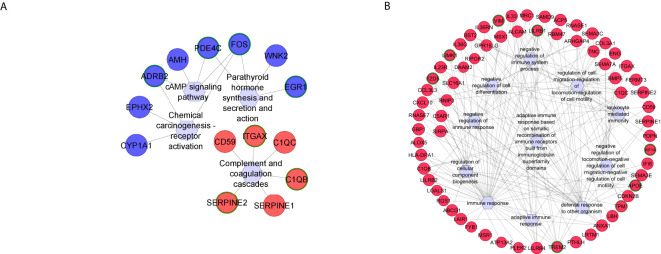
Functional networks of selected **(A)** KEGG pathways and **(B)** GO biological processes created using differentially expressed genes specific to *A. flavus* keratitis. Downregulated and upregulated genes are marked in blue and red, respectively. The hub genes are marked in green in outline. Hexagonal shapes represent the pathways or GO.

Four KEGG pathways were identified using *A. flavus-*specific DEGs, which were activated (**Supplementary Table S3**), with three hub genes identified. Suppressed pathways, including parathyroid hormone synthesis, cyclic adenosine monophosphate (cAMP) signaling, and chemical carcinogenesis-receptor activation, were also notable, with the fos proto-oncogene, AP-1 transcription factor subunit (FOS) as a central hub gene across these pathways (**Figure 5A**).

GO analysis of *A. flavus-*specific genes revealed 145 biological pathways (**Supplementary Table S4**). The top activated GO terms include adaptive immune response, regulation of cell migration, and regulation of cell motility, while suppressed pathways include epidermal cell differentiation and regulation of cell cycle process (**Figure 5B**). The adaptive immune response, negative regulation of immune system process, and negative regulation of immune response enriched with several genes have key hub genes such as triggering receptor expressed on myeloid cells 2 (TREM2), leukocyte immunoglobulin like receptor B4 (LILRB4), leukocyte immunoglobulin like receptor B4 (LILRB1), and guanylate binding protein 1 (GBP1) (**Figure 5B**). Regulation of cell migration, negative regulation of cell migration, and negative regulation of cell motility were activated and had hub genes apolipoprotein E (APOE) and TREM2, while the transcription regulation pathway was suppressed and had hub genes chromobox 4 (CBX4) and SRY-box transcription factor 15 (SOX15) (**Figure 5B**).

### 3.5 Confirmation of DEGs using RT-qPCR

**Figure 6:**
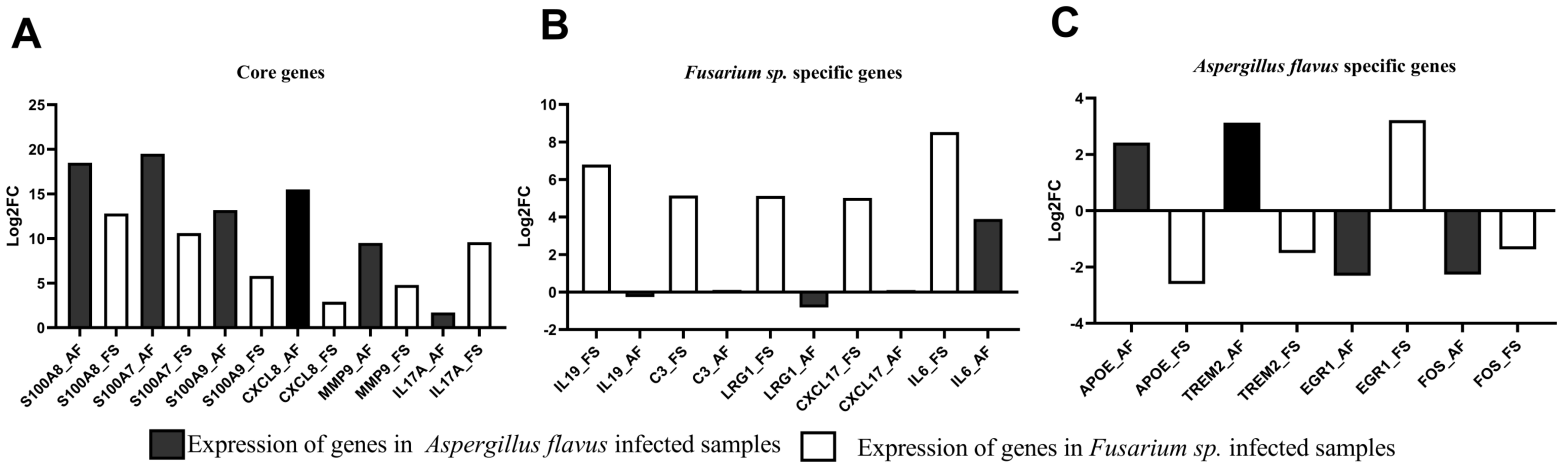
Differential expression analysis of selected genes in *Fusarium* spp.- and *A. flavus*-infected tissue samples compared to cadaver controls using RT-qPCR. The log2 fold change (Log2FC) values were plotted against selected **(A)** common differentially expressed genes (DEGs), **(B)** *Fusarium* spp.-specific DEGs, and **(C)** *A. flavus*-specific DEGs.

RT-qPCR analysis was performed on 14 selected hub genes (**Supplementary Figure S3**) identified through mRNA sequencing. In addition, IL-17A was selected as it is the activator of the IL-17 signaling pathway. Key findings include the upregulation of S100A7, S100A8, S100A9, CXCL8, and interleukin 17A (IL-17A) in both *A. flavus* and *Fusarium* spp. keratitis samples (**Figure 6A**; **Supplementary Figure S3A**), consistent with mRNA sequencing results. Significant upregulation of interleukin 19 (IL-19), C3, and LRG1 were detected in *Fusarium* spp. keratitis and TREM2 and APOE in *A. flavus* keratitis (**Figures 6B, C**; **Supplementary Figures S3B, C**). The *Fusarium* spp.-specific DEG, IL-6, and *A. flavus*-specific DEGs, FOS, and Early Growth Response 1 (EGR1), identified through mRNA sequencing, did not show concordance with RT-qPCR. Nevertheless, these results demonstrate the distinct immune activation profiles for each pathogen.

## 4 Discussion

This study highlights key inflammatory pathways enriched in both *A. flavus* and *Fusarium* spp. keratitis, including IL-17 signaling, NF-kappaB signaling, TNF signaling, and cytokine–cytokine receptor interaction, as central to the immune response of the host. These pathways modulate the host’s defense mechanisms and contribute to inflammation and disease modulation. The upregulation of genes like S100A8/A9, CXCL8, and IL-1B underscores a robust inflammatory response, consistent with previous findings (Monin and Gaffen, 2018). While IL-17 signaling enhances epithelial barrier function and promotes antimicrobial peptides, its excessive activation in corneal tissue was linked to poor outcomes in our study. Karthikeyan et al. (2011) also showed a significant expression of IL-17 in both early and late stages of FK caused by *A. flavus* and *Fusarium* spp., suggesting a crucial role in the IL-17 signaling pathway. This aligns with reports suggesting that modulating IL-17 can mitigate hyperinflammation in severe FK (Wang et al., 2018; Qin et al., 2019). CXCL8, a chemokine involved in neutrophil recruitment, was significantly upregulated in both *A. flavus* and *Fusarium* spp. infections, further emphasizing the critical role of neutrophils in FK pathogenesis. Its expression by corneal epithelial cells and immune cells during fungal infections has been reported previously (Zhou et al., 2023; Alenezi et al., 2024). In our analysis, CXCL8 expression was higher in *A. flavus* than in *Fusarium* spp., consistent with earlier studies (Arunachalam et al., 2022). Overall, our findings underscore the dual role of inflammatory pathways in FK pathogenesis, with IL-17 and CXCL8 serving as key mediators of both immune defense and disease progression. Balancing these responses may offer novel therapeutic opportunities.

Furthermore, the study identified *A. flavus*- and *Fusarium* spp.-specific DEGs and subsequent functional enrichment, highlighting distinctive immune responses. Notably, the *Fusarium* spp.-specific DEG C3 was enriched in pathways such as the acute inflammatory response and the positive regulation of the immune response. Tear protein analysis revealed that, unlike samples from patients with *A. flavus* keratitis, tears from *Fusarium* spp. keratitis patients exhibited slightly higher levels of C3 and its cleaved products (Shait Mohammed et al., 2020). In this study, C3 was also observed as specific to *Fusarium* spp. keratitis and not significantly expressed in *A. flavus* keratitis compared to the control (**Figures 6B, C**; **Supplementary Figure S3B**). In the KEGG enrichment analysis, C3 is enriched in neutrophil extracellular trap formation pathway. However, Kandhavelu et al. (2017) reported the role of Calprotectin (S100A8/S100A9) in the clearance of *A. flavus* hyphae via neutrophil extracellular trap formation; in this study, S100A8 and S100A9 were identified as common DEGs. Nevertheless, this study confirms that C3 is a marker for *Fusarium* spp. keratitis, distinguishing it from *A. flavus* keratitis. This further suggests that targeting complement pathways could be a novel approach to managing *Fusarium* spp. keratitis.

mBD3 was differentially expressed in mice cornea (Kolar et al., 2013); FHL and zinc-alpha-2 glycoprotein were differentially expressed in the tears of patients with keratitis infected by *A. flavus* and *Fusarium* spp (Shait Mohammed et al., 2020; Ananthi et al., 2013; Parthiban et al., 2019). However, we did not find such differences in our dataset. Indeed Shait Mohammed et al. (2020) reported the absence of FHL in the tears of patients infected with *Fusarium* spp., while in our study, Complement Factor H (CFH) encoding FHL was insignificantly downregulated with a fold change of -1.4 and thus filtered (see “Materials and methods”). The possible reasons include that we used total RNA from post-TPK corneal tissues for differential gene expression, while other studies used tears and the mice cornea. Furthermore, post-transcriptional regulatory mechanisms affect the concordance between protein and mRNA expression (Vélez-Bermúdez and Schmidt, 2014).

The unique expression of IL-19 enriched in the immune response pathway in *Fusarium* spp. keratitis, which has not been previously reported, suggests that it could be a specific marker for this infection. This discovery, supported by RT-qPCR (**Figure 6B**; **Supplementary Figure S3B**), adds to our understanding of the cytokine network in *Fusarium* spp. infections. Moreover, the enriched presence of LRG1 in *Fusarium* spp. keratitis (**Figure 6B**; **Supplementary Figure S3B**), confirmed by RT-qPCR, suggests its involvement in immune regulation and leukocyte adhesion, which is critical for the resolution of inflammation and tissue repair during infection (Camilli et al., 2022). Interestingly, in normal individuals, the expression of LRG1 appears to be substantially higher in the Indian population compared to the United Kingdom population (Gurudas et al., 2022). The role of LRG1 in immune cell regulation further emphasizes the complexity of host–pathogen interactions in fungal keratitis, as it could potentially influence both disease progression and therapeutic responses.

In *A. flavus* keratitis, pathogen-specific hub DEGs such as TREM2, LILRB4, LILRB1, and GBP1 had enriched adaptive immune response, negative regulation of immune system process, and negative regulation of immune response. Of these, TREM2, confirmed by RT-qPCR, was reported to promote host resistance to bacterial infection by suppressing corneal inflammation (Sun et al., 2013 et al., Wu et al., 2024). Furthermore, *A. flavus*-specific DEG APOE, which is involved in cell migration and motility and was confirmed by RT-qPCR (**Figure 6C**; **Supplementary Figure S3C**), has been studied as a marker for corneal involvement in acute infectious conjunctivitis (Seitzman et al., 2024). However, the role of TREM2 and APOE during *A. flavus* keratitis has yet to be studied.

The patients in this study were treated with different antifungal regimens (**Supplementary Table S1**). While all received natamycin, some were also administered voriconazole, econazole, or itraconazole. According to the MUTT study (Venkatesh Prajna et al., 2013), natamycin is the preferred treatment for *Fusarium* spp. keratitis, while *A. flavus* infections may be treated with either natamycin or voriconazole. This pattern is reflected in our dataset (**Supplementary Table S1**), where *Fusarium* spp. cases (FK01–FK10) were primarily treated with natamycin, whereas some *A. flavus* cases (AFK01–AFK10) received voriconazole in addition to natamycin. Despite these treatment differences, our transcriptomic data (**Figure 1**; **Supplementary Figure S2**) and RT-qPCR analyses demonstrated clear pathogen-specific differences in host response—for instance, FK05 treated with natamycin along with voriconazole was clearly segregated from the *A. flavus* group in the PCA plot (**Supplementary Figure S2C**). We show that the major transcriptomic differences corresponded to pathogen type rather than treatment regimen, indicating that host immune responses were primarily driven by the infecting fungal species rather than the antifungal drugs administered. Further studies investigating treatment-induced changes and protein-level expression are warranted.

The differences in corneal responses to *Fusarium* spp. and *A. flavus* infections can be attributed to distinct pathogen-specific virulence factors, structural components, and interactions with the host immune system. *Fusarium* spp. produce a variety of mycotoxins, notably trichothecenes, fumonisins, and fusaric acid, which directly modulate host immune responses and induce cellular apoptosis (Brown et al., 2012; Guarro, 2013; Keller, 2019). In contrast, *A. flavus* is characterized by the production of aflatoxins, known for their potent immunosuppressive effects (Lionakis and Kontoyiannis, 2003; Yoshimi et al., 2016). Furthermore, according to a review by Abbondante et al. (2023), *Fusarium oxysporum* possesses lineage-specific pathogenicity chromosomes that are distinct from their core chromosomes and are thought to contribute to host-specific pathogenicity. These chromosomes carry genes that may encode unique virulence factors, enabling them to survive and cause disease in the human cornea, potentially leading to immune responses that differ from those elicited by *Aspergillus*, which lacks these specific chromosomal features (Abbondante et al., 2023). Structurally, *Fusarium* spp. exhibit variability in their cell wall components, particularly β-glucans and mannans, leading to distinct patterns of host pattern recognition receptor (PRR) activation (Gow et al., 2017; Abbondante et al., 2023). *A. flavus*, on the other hand, possesses a cell wall rich in β-glucans, galactomannan, and chitin (Yoshimi et al., 2016; Abbondante et al., 2023). Additionally, *Fusarium* spp. exhibits rapid germination and invasive hyphal growth, which facilitates effective immune evasion (Osherov and Yarden, 2010). In contrast, *A. flavus* employs a strategy of modulating host immune pathways by masking key immunogenic components (Abbondante et al., 2023). These varying fungal traits, including mycotoxin profiles, cell wall composition, and growth strategies, may highlight the unique transcriptomic profiles identified in our study and may contribute to the differing inflammatory responses seen in fungal keratitis.

This study has some limitations. First, very few samples were used for transcriptomic analysis. Although we validated some key genes using RT-qPCR, validation of all the key pathway genes in a large cohort of samples is warranted. Next, the identification of fungal species using molecular methods was not performed in this study. Lastly, though we could identify pathogen-specific differences in host response at the mRNA level, further studies investigating treatment-induced changes and protein-level expression are required.

## 5 Conclusion

This study identifies pathogen-specific and pathogen-independent host immune responses triggered by *Fusarium* spp. and *A. flavus* in fungal keratitis. Pathway enrichment analysis revealed common immune pathways, such as IL-17, TNF, and chemokine signaling, shared by both fungal infections. *Fusarium* spp.-specific DEGs, including C3, IL-6, IL-19, and LRG1, are enriched in pathways such as positive regulation of immune responses, acute inflammatory responses, leukocyte cell–cell adhesion, and the regulation of cell–cell adhesion. *A. flavus*-specific DEGs, such as TREM2 and APOE, are predominantly enriched in adaptive immune response, cell migration, and motility pathways, respectively. These findings enhance our understanding of immune dynamics in fungal keratitis and provide a foundation for future research, potentially informing more effective therapeutic strategies.

## Data Availability

The original contributions presented in the study are publicly available. This data can be found here: NCBI / PRJNA1171184.

## Supplementary material

Supplementary Figure 1 Identifying hub genes by protein–protein interaction (PPI) network created using the genes of the significant modules from the WGCNA analysis (see “Materials and methods”). **(A)** PPI network of common genes, **(B)** *Fusarium* spp.-specific genes, and **(C)** *A. flavus*-specific genes. The identified hub genes are highlighted in yellow to red.

Supplementary Figure 2 Principal component analysis (PCA) to identify the clustering of control and *A. flavus*- and *Fusarium* spp.-infected sample group based on the first two principal components (PC1 and PC2). PCA plot showing **(A)** clustering of control samples (red) and *Fusarium* spp.-infected samples (blue), **(B)** clustering of control samples (red) and *A. flavus*-infected samples (green), and **(C)** clustering of *Fusarium* spp.-infected (blue) and *A. flavus*-infected samples (green).

Supplementary Figure 3 Expression of selected genes in *A. flavus*- and *Fusarium* spp.-infected samples compared to cadaver control samples. Delta Ct (-dct) value of selected **(A)** commonly and **(B)** *Fusarium* spp.-specific and **(C)** *A. flavus*-specific dysregulated genes in *A. flavus*- and *Fusarium* spp.-infected samples. The statistical significance was calculated using the non-parametric Mann–Whitney test.
