# Supplementary figures and images for: Fusarium spp. and Aspergillus flavus infection induces pathogen-specific and pathogen-independent host immune response in patients with fungal keratitis
Source: Front Cell Infect Microbiol. 2025 May 30;15:1560628. doi: 10.3389/fcimb.2025.1560628 (PMC12162692; doi:10.3389/fcimb.2025.1560628)

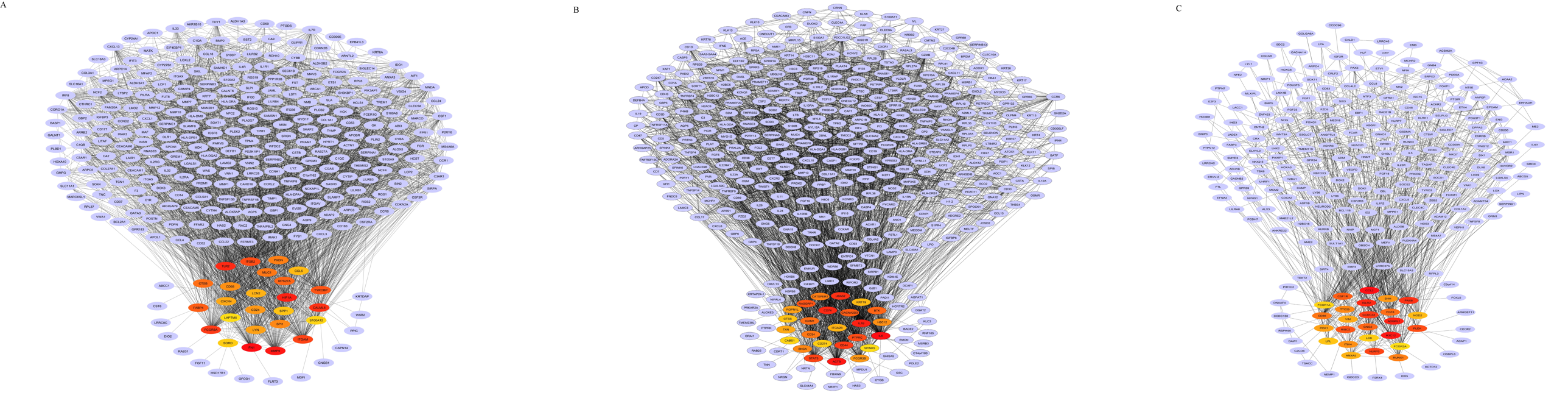

Supplement: Supplementary Figure 1 — Identifying hub genes by protein–protein interaction (PPI) network created using the genes of the significant modules from the WGCNA analysis (see “Materials and methods”). (A) PPI network of common genes, (B) Fusarium spp.-specific genes, and (C) A. flavus-specific genes. The identified hub genes are highlighted in yellow to red. [file Image1.jpeg]
