# Supplementary material for: Fusarium spp. and Aspergillus flavus infection induces pathogen-specific and pathogen-independent host immune response in patients with fungal keratitis
Source: Front Cell Infect Microbiol. 2025 May 30;15:1560628. doi: 10.3389/fcimb.2025.1560628 (PMC12162692; doi:10.3389/fcimb.2025.1560628)
Supplement: Supplementary file 4 [file Table1.docx]

| **Supplementary Table 1: Details of the samples used for sequencing and RT-qPCR** | | | | | | | | | | | |
| --- | --- | --- | --- | --- | --- | --- | --- | --- | --- | --- | --- |
| **Patient ID** | **Age/**  **Gender** | **Duration of symptom (days)** | **Characteristics of ulcer** | | **Hypopyon presence** | **Hypopyon size (mm)** | **Treatment** | **Time of surgery (TPK*) after first visit (days)** | **Visual acuity**  **(Initial)** | **Visual acuity (Final)** | **Graft status** |
|  |  |  | **Size (mm^2^)** | **Depth** |  |  |  |  |  |  |  |
| FK01 | 32/F | 7 | 6x8 | Normal | Y | 1.5 | Natamycin  Moxifloxacin  Econozole | 14 | 5/60 | HM | Failed graft |
| FK02 | 65/F | 14 | 1x1 | 70% | N | - | Fluconozole,  Gatifloxacin  Homatropine  Natamycin  Moxifloxacin  Homatropine  paracetamol | 10 | 5/60 | LP+ | Failed graft |
| FK03 | 37/M | 3 | 4x4 | 40% | Y | Streak Hypopyon | Natamycin  Econozole  Atropine Sulphate Paracetamol | 14 | 2/60 | HM | Failed graft |
| FK04 | 58/F | 3 | 5x5 | 33% | Y | Streak Hypopyon | Natamycin  Homatropine  Itraconozole Paracetamol | 60 | LP+ | HM | Failed graft |
| FK05 | 65M | 10 | 8x5 | - | Y | 0.75 | Natamycin  Voriconozole Atropine Sulphate Paracetamol | 21 | LP+ | LP+ | Clear graft |
| FK06 | 57/F | 10 | 4x4.5 | 40% | Y | 2 | Natamycin  Econozole Atropine Sulphate Paracetamol | 14 | HM | LP+ | Clear graft |
| FK07 | 68/M | 7 | 8x8 |  | Y | 6 | Natamycin  Econozole Atropine Sulphate Itraconozole Paracetamol, Pantoprazole | 7 | LP+ | HM | Failed graft |
| FK08 | 50/M | 3 | 6.5 | 60% | Y | 6 | Natamycin  Voriconozole Atropine Sulphate Paracetamol  Pantoprazole | 10 | HM | HM | Failed graft |
| FK09 | 37/F | 7 | 6.5x6 | 50% | Y | 6.5 | Natamycin  Voriconozole Homatropine | 14 | HM | 1/60 | Failed graft |
| FK10 | 51/M | 10 | 5x5 |  | N | - | Natamycin  Econozole Atropine Sulphate Paracetamol | 2 | LP+ | - | Edematous graft |
| AFK01 | 60/M | 7 | 7*6 | 70% | N | - | Natamycin  Econozole  Atropine Sulphate Paracetamol | 7 | LP+ | LP+ | Failed graft |
| AFK02 | 64/M | 20 | 6x6 | 30% | Y | 2 | Natamycin  Econozole Atropine Sulphate Paracetamol | 7 | LP+ | HM | Clear graft |
| AFK03 | 40/M | 7 | 3.7x2.9 | 33% | Y | Trace hypopyon | Natamycin  Econozole Atropine Sulphate Paracetamol | 30 | 5/60 | HM | Failed graft |
| AFK04 | 55/M | 7 | 6.4x8 | 30% | Y | 3.5 | Natamycin  Econozole Atropine Sulphate Paracetamol | 21 | HM | HM | Clear graft |
| AFK05 | 64/M | 11 | 6*5 | 33% | Y | 1.5 | Natamycin  Voriconozole Atropine Sulphate Paracetamol | 21 | HM | HM | Failed graft |
| AFK06 | 56/M | 3 | 2*2 | 33% | N | - | Natamycin Moxiflaxacin  Itraconozole Homatropine | 60 | HM | HM | Failed graft |
| AFK07 | 56/M | 14 | 4.5x4.1 | 50% | Y | 2.2 | Natamycin  Econozole Atropine Sulphate Paracetamol | 14 | FCF | FCF | Failed Graft |
| AFK08 | 44/M | 2 | 5x5 | 20% | Y | Trace hypopyon | Natamycin Voriconozole Homatropine Paracetamol pantoprazole | 3 | 6/60 | LP+ | Failed graft |
| AFK09 | 66/M | 3 | 5x4 | - | N | - | Natamycin  Atropine Sulphate Paracetamol | 30 | 6/60 | HM | Edematous graft |
| AFK10 | 66/F | 7 | 8x7 | 75% | Y | 5 | Natamycin  Voroconozole Homatropine  Paracetamol pantoprazole | 1 | LP+ |  | Failed graft |
| CC01 | 53/M | - | - | - | - | - | - | - | - | - | - |
| CC02 | 64/M | - | - | - | - | - | - | - | - | - | - |
| CC03 | 66/F | - | - | - | - | - | - | - | - | - | - |
| CC04 | 43/M | - | - | - | - | - | - | - | - | - | - |
| CC05 | 78/M | - | - | - | - | - | - | - | - | - | - |
| CC06 | 77/M | - | - | - | - | - | - | - | - | - | - |
| CC07 | 68/M | - | - | - | - | - | - | - | - | - | - |
| CC08 | 64/M | - | - | - | - | - | - | - | - | - | - |
| CC09 | 65/M | - | - | - | - | - | - | - | - | - | - |
| CC10 | 80/F | - | - | - | - | - | - | - | - | - | - |
| CC11 | 85/F | - | - | - | - | - | - | - | - | - | - |
| CC12 | 40/F | - | - | - | - | - | - | - | - | - | - |
| CC13 | 57/M | - | - | - | - | - | - | - | - | - | - |
| CC14 | 86/F | - | - | - | - | - | - | - | - | - | - |
| CC15 | 90/M | - | - | - | - | - | - | - | - | - | - |
| CC16 | 31/M | - | - | - | - | - | - | - | - | - | - |
| CC17 | 22/M | - | - | - | - | - | - | - | - | - | - |
| CC18 | 35/M | - | - | - | - | - | - | - | - | - | - |
| CC19 | 97/M | - | - | - | - | - | - | - | - | - | - |
| CC20 | 27/M | - | - | - | - | - | - | - | - | - | - |

**(HM - Hand movement, LP+ - Light perception, FCF - Finger counting close to face)**
